# Maternal Use of Antibiotics, Hospitalisation for Infection during Pregnancy, and Risk of Childhood Epilepsy: A Population-Based Cohort Study

**DOI:** 10.1371/journal.pone.0030850

**Published:** 2012-01-25

**Authors:** Mette Nørgaard, Vera Ehrenstein, Rikke Beck Nielsen, Leiv Sigmund Bakketeig, Henrik Toft Sørensen

**Affiliations:** 1 Department of Clinical Epidemiology, Institute of Clinical Medicine, Aarhus University Hospital, Aarhus, Denmark; 2 Norwegian Institute of Public Health, Nydalen, Norway; Columbia University, United States of America

## Abstract

**Background:**

Maternal infection during pregnancy may be a risk factor for epilepsy in offspring. Use of antibiotics is a valid marker of infection.

**Methodology/Principal Findings:**

To examine the relationship between maternal infection during pregnancy and risk of childhood epilepsy we conducted a historical cohort study of singletons born in northern Denmark from 1998 through 2008 who survived ≥29 days. We used population-based medical databases to ascertain maternal use of antibiotics or hospital contacts with infection during pregnancy, as well as first-time hospital contacts with a diagnosis of epilepsy among offspring. We compared incidence rates (IR) of epilepsy among children of mothers with and without infection during pregnancy. We examined the outcome according to trimester of exposure, type of antibiotic, and total number of prescriptions, using Poisson regression to estimate incidence rate ratios (IRRs) while adjusting for covariates. Among 191 383 children in the cohort, 948 (0.5%) were hospitalised or had an outpatient visit for epilepsy during follow-up, yielding an IR of 91 per 100 000 person-years (PY). The five-year cumulative incidence of epilepsy was 4.5 per 1000 children. Among children exposed prenatally to maternal infection, the IR was 117 per 100 000 PY, with an adjusted IRR of 1.40 (95% confidence interval (CI): 1.22–1.61), compared with unexposed children. The association was unaffected by trimester of exposure, antibiotic type, or prescription count.

**Conclusions/Significance:**

Prenatal exposure to maternal infection is associated with an increased risk of epilepsy in childhood. The similarity of estimates across types of antibiotics suggests that processes common to all infections underlie this outcome, rather than specific pathogens or drugs.

## Introduction

Epilepsy is the most common serious neurologic disorder of childhood [1]–[3]. Before the age of 15 years, 1.0%–1.7% of all children will have at least one unprovoked seizure and up to 0.8% will have repeated seizures [2]. The incidence of childhood epilepsy is highest in the first year of life – about 150 per 100 000 person-years (PY), falling to around 50 per 100 000 PY after age 9 years [2].

The aetiology of epilepsy is poorly understood [1], [2], with no known risk factor in many cases [1], [4], [5]. Generalised epilepsies often can be traced infection [2]. In particular, insults acting during the prenatal and to single-gene mutations or chromosomal abnormalities, while partial epilepsies are frequently triggered by external insults to the central nervous system, including brain injury and central nervous system neonatal period are thought to contribute to the aetiology of some epilepsies [1], [2], [6]–[8].

Observation of a seasonal pattern, *i.e.* an excess of births during winter months among children who develop epilepsy [9], [10], led to the suggestion that prenatal exposure to maternal infection may be a risk factor. In a Canadian population-based study of 648 new cases of epilepsy among 124 207 children born in 1986–2000 and followed for a median of 8.5 years, any maternal infection during pregnancy (method of measurement unspecified) was associated with a 1.0–1.6-fold increase in risk of childhood epilepsy [8]. In a previous Danish study, maternal self-reported history of cystitis, pyelonephritis, vaginal yeast infection, and/or symptoms of infection (diarrhoea, coughs) during pregnancy was associated with a 1.2- to 2.6-fold increased risk of epilepsy among offspring [11]. Risk varied by type of infection and was the highest for self-reported vaginal yeast infection (incidence rate ratio (IRR) = 2.6 (95% CI: 1.4–4.6)) and pyelonephritis (IRR = 2.3 (95% CI: 1.2–4.7)) [11].

We conducted a large population-based historical cohort study in Denmark to examine the relationship between maternal bacterial or fungal infection during pregnancy and risk of childhood epilepsy in singletons born from 1998 through 2008. In contrast to the earlier study from Denmark that relied on self-report, we used routinely recorded markers of infection – prescription and hospitalisation records – to examine the role of the type and severity of infection and trimester of exposure. Furthermore, we examined whether the association between infections and epilepsy differed by maternal history of epilepsy.

## Methods

### Setting

We conducted this study in the Central and the Northern Regions of Denmark, with a combined population of 1.8 million persons. The Danish National Health Service provides tax-supported healthcare, with free access to hospital-based and primary medical care and partial reimbursement for the costs of most prescribed drugs, including antibiotics.

### Study population

From the Danish Medical Birth Registry [12], we retrieved birth records of all singletons born in the two regions during the 1998–2008, who survived at least 29 days [11]. We imposed this survival requirement to allow time for diagnostic work-up. The Birth Registry contains computerised records of all births in Denmark since 1973. Data are collected by midwives or physicians overseeing the deliveries and include the civil registration numbers of the mother and the newborn, permitting linkage to maternal records.

### Data linkage

The unique ten-digit civil registration number, assigned to all residents of Denmark by the Central Office of Civil Registration since 1968 [13], is used in all Danish registries, permitting unambiguous individual-level linkage of data from all sources used in this study.

### Maternal infection during pregnancy

We measured prenatal exposure to maternal infection by ascertaining maternal antibiotic prescriptions and maternal history of inpatient hospital stays or outpatient/emergency visits with a diagnosis of infection. The Northern and Central Regions of Denmark are served by pharmacies equipped with electronic accounting systems used primarily to secure reimbursement from the National Health Service. Prescription information, including the customer's civil registration number, the type and amount of drug prescribed according to the Anatomical Therapeutic Chemical (ATC) classification system, and the date of dispensation, is transferred from the pharmacies to the Aarhus University Prescription Database [14]. All filled reimbursed prescriptions are included in this database, including prescriptions from doctors working in hospitals or private practising doctors. We used this database to identify all prescriptions for systemic antibiotics and systemic or vaginal antifungals, filled from the estimated date of conception until birth (see Appendix S1 for relevant ATC codes). Because drugs administered during hospitalisations do not generate records in the prescription database, we retrieved from the Danish National Registry of Patients (DNRP), maternal diagnoses of infection recorded between the estimated conception date and birth. The DNRP has tracked hospitalisations since 1977 and outpatient and emergency room visits at all public hospitals in Denmark since 1995 [15]. The recorded data include patients' civil registration numbers, dates of admission and discharge, and up to 20 diagnoses, classified according to the *International Classification of Diseases*, 10th revision (ICD-10). We included all hospital contacts (emergency and inpatient admissions, and outpatient visits) with a diagnosis of infection. The relevant ICD-10 codes are listed in Appendix S1. Newborns whose mothers had a record of either an antibiotic prescription filled at a community pharmacy or a hospital visit with a diagnosis of infection were considered prenatally exposed to a maternal infection. Newborns without this maternal history were used as the reference group for all comparisons in this study.

We categorised prescribed antibiotics into penicillins, pivmecillinam, sulfamethizole and/or trimethoprim, macrolides, antifungals and all other antibiotics. In Denmark, pivmecillinam and sulfamethizole and/or trimethoprim are used predominantly for urinary tract infections and penicillin is used primarily for respiratory infections [16].

### Epilepsy

As nearly all children with epilepsy in Denmark are expected to have a hospital contact (admission or outpatient clinic visit) [17], we used the DNRP to identify children with this diagnosis. We defined the study outcome as a first-time hospitalisation or an outpatient visit with a diagnosis of epilepsy recorded in the DNRP during the follow-up period. We also ascertained children's prescriptions for antiepileptic drugs using the ATC code N03. We did not use prescription records to define childhood epilepsy because physicians may refrain from or postpone antiepileptic drug treatment in about 15% of new-onset cases [18].

### Covariates

We identified potential confounding factors based on our *a priori* knowledge on potential risk factors for childhood epilepsy that could also be associated with maternal infection. We defined maternal epilepsy [19] as a prescription for an antiepileptic drug filled at any time from conception until giving birth or a ICD-10 code for epilepsy recorded in DNRP (See Appendix S1 for relevant ATC and ICD-10 codes). We similarly defined maternal diabetes as a prescription for an antidiabetic filled at any time before or during gestation of a child in the study population or an ICD-10 code for diabetes [8], [19] recorded in the DNRP (See Appendix S1 for relevant ATC and ICD-10 codes). From the DNRP we obtained history of maternal hospitalisation with preeclampsia or eclampsia [8], [20] during the relevant pregnancy. From the Medical Birth Registry, we obtained information on birth order [8], maternal age [8], [19], self-reported smoking during pregnancy [8], caesarean delivery [8], gestational age [7], [21], birth weight [7], and Apgar score at five minutes [6], [22]. Furthermore, because congenital malformations are associated with an increased risk of epilepsy [23], [24] and may also be associated with maternal use of antibiotics [25], we ascertained from the DNRP all diagnoses of congenital malformations detected within the first year of life among the children in the study population. See Appendix S1 for relevant codes.

### Statistical analysis

We followed each child from the 29^th^ day of life until the date of the first epilepsy-related hospitalisation/outpatient contact, emigration, death, or 1 January 2009, whichever came first. We computed incidence rates (IR) of epilepsy overall and according to prenatal exposure to maternal infection as defined in this study (overall and in each trimester). We computed IRs for the first, second, third, fourth and subsequent years of life and estimated the one- and five-year cumulative incidence of epilepsy using the formula (cumulative incidence = 1-exp(-IR×*t*), where *t* indicates time), assuming constant average IR [26]. We categorised examined the total number of prescriptions for antibiotics dispensed during pregnancy (1, 2, 3, >3), as a marker of severity, to evaluate a potential dose-response pattern, using the χ^2^ test for trend. In addition to examining potential variation according to maternal history of epilepsy, we examined whether the association between maternal infection and epilepsy varied according to a child's sex, birth order, maternal age at delivery, smoking during pregnancy, maternal history of diabetes or preeclampsia/eclampsia (one variable), caesarean delivery, gestational age, being small for gestational age (SGA) at term, and Apgar score at five minutes. We used Poisson regression to estimate IRRs for epilepsy-related hospitalisations/outpatient and emergency contacts associated with maternal infection, while adjusting for birth order(firstborn, yes/no), calendar year of birth (annual indicator for each year between 1998 and 2008 and in the time intervals 1998–2002 vs. 2003–2008 for analyses restricted to children of mothers with epilepsy), maternal age (≤20, 21–34, ≥35 years), smoking in pregnancy (yes/no), history of epilepsy (yes/no), or history of diabetes or preeclampsia/eclampsia (yes/no). In an additional analysis we estimated IRRs for partial epilepsy and generalised epilepsy separately. Partial epilepsy was defined as an ever-recorded diagnosis of partial epilepsy and no diagnosis of generalised epilepsy. Generalised epilepsy was defined as an ever-recorded diagnosis of generalised epilepsy in the absence of a history of partial epilepsy. Children with diagnoses for both types of epilepsy and those who only had unspecified epilepsy were excluded from this analysis.

In a series of sensitivity analyses, we examined the effect of additional adjustment for low birth weight (<2500 g) and preterm birth (<37 weeks), the effect of restriction to children without congenital malformations, and the effect of using dispensing of antiepileptic drugs to define the outcome of epilepsy in children.

We used SAS version 9.02 (SAS Institute Inc., Cary, North Carolina, USA). The study was approved by the Danish Registry Board (Record no. 2002–41–1820).

### Role of the funding source

The funding source had no role in the study design, data collection, data analysis, data interpretation, or writing of the paper. All authors had full access to all the data in the study and HTS had the final responsibility for the decision to submit for publication.

## Results

Among 191 383 eligible singleton births, 57 826 (30%) newborns were prenatally exposed to maternal infection. Maternal antibiotic use was recorded for 55 743 (29%) newborns and maternal hospital contacts with infection were recorded for 6292 (3%). In total 4209 (2%) children had a record of a maternal prescription for antibiotics and of a hospital contact with an infection during the gestation. The children were followed for a median of 5 years and up to 11 years. Cumulatively, there were 1 041 843 PY of observation. Table 1 presents baseline characteristics of pregnancies according to maternal infection status in pregnancy.

**Table 1:** Descriptive data on 191 383 singleton births in the Northern and Central Regions of Denmark during 1998–2008, according to maternal use of antibiotics or hospitalisations with infections during pregnancy.

	Prenatal Exposure to Infection				Total	
	No		Yes			
	N	%	N	%	N	%
Total	133 557	70·0	57 826	30·0	191 383	100·0
Maternal prescription for antibiotics during pregnancy	0	0	55 743	96·4	55 743	29·1
Inpatient or outpatient hospital visit with infection	0	0	6 292	10·9	6 292	3·3
Maternal age at delivery, years						
≤20	2 884	2·2	1 980	3·4	4 864	2·5
21–34	109 933	82·3	47 074	81·4	157 007	82·0
≥35	20 740	15·5	8 772	15·2	29 512	15·4
Firstborn	75 043	56·2	35 982	62·2	111 025	58·0
Maternal smoking in pregnancy	22 526	16·9	12 851	22·2	35 377	18·5
Maternal history of epilepsy	1 289	1·0	788	1·4	2 077	1·1
Maternal history of diabetes	1 093	0·8	854	1·5	1 947	1·0
Preeclampsia in relevant pregnancy	3 194	2·4	1 675	2·9	4 824	2·5
Cesarean delivery	21 261	15·9	10 282	17·8	31 543	16·5
Sex						
Girl	65 119	48·8	28 139	48·7	93 258	48·7
Boy	68 438	51·2	29 687	51·3	98 125	51·3
Birth weight <2500 grams	4 251	3·2	2 001	3·5	6 252	3·3
Gestation <37 weeks	6 253	4·7	3 046	5·3	9 299	4·9
Apgar <7 at 5 minutes	784	0·6	347	0·6	1 131	0·6
Malformation	4 828	3·6	2 244	3·9	7 072	3·7

During the follow-up, 948 infants had a hospital contact for epilepsy (718 inpatient admissions (76%) and 230 outpatient clinic visits (24%)), corresponding to an overall IR of 91 per 100 000 PY. The IR was highest in the first year of life (193 per 100 000 PY based on 332 epilepsy cases). IRs (per 100 000 PY) during the 2nd, 3rd, and 4th–11th years of life were 96, 68, and 64, respectively. The one-year cumulative incidence of epilepsy was 1·9 per 1000 children; the five-year cumulative incidence was 4·5 per 1000 children.

The IR for epilepsy was higher among singletons prenatally exposed to maternal infection (overall IR = 117 per 100 000 PY); adjusted incidence rate ratio (IRR) = 1.40 (95% CI: 1.22–1.61) compared with unexposed singletons. IRs were similar among children prenatally exposed to different subgroups of antibiotics (Table 2). The IRR of epilepsy did not differ substantially either by trimester of exposure or by the length of follow-up (Table 3). The adjusted IRR associated with maternal hospital admissions with infection was 1.36 (95% CI: 0.97–1.90). We observed a higher risk of epilepsy with an increasing total number of antibiotic prescriptions, suggesting a dose-response pattern (Table 3). A maternal record of an antifungal prescription was present for 19 019 (9.9%) of the children, with 93% of cases associated with maternal use of vaginal antifungals. The incidence rate of epilepsy among children exposed to maternal antifungals was 88 per 100 000 PY, and the associated adjusted IRR was 0.98 (95% CI: 0.78–1.20) compared with the unexposed. All estimates remained virtually unchanged after additional adjustment for preterm birth and low birth weight (data not shown).

**Table 2:** Incidence rates of epilepsy (per 100 000 person-years) and adjusted incidence rate ratios overall and according to type of prenatal antibiotic exposure within 30 days before conception or during pregnancy.

Antibiotic use	Births, N	Epilepsy cases, N	Person-years	Incidence rate	Adjusted* incidence rate ratio (95% CI)
None	131 307	587	730 657	8·0	1·0
Any	60 076	361	311 176	11·6	1·40 (1·22–1·61)
Pivmecillinam	17 756	106	76 527	13·9	1·55 (1·25–1·93)
Penicillin V	27 150	177	145 012	12·2	1·56 (1·30–1·87)
Other penicillins	12 259	79	65 755	12·0	1·46 (1·15–1·87)
Sulfonamides/trimethoprim	12 748	83	67 782	12·2	1·42 (1·12–1·82)
Macrolides	5 847	46	33 697	13·7	1·61 (1·15–2·25)

*Adjusted for birth year, birth order (1, 2+), maternal age (≤20, 21–34, ≥35), smoking in pregnancy (yes/no), maternal history of epilepsy, and maternal history of diabetes or preeclampsia/eclampsia.

**Table 3:** Crude and adjusted incidence rate ratios with 95% confidence intervals (CI) for epilepsy diagnosed in the entire follow-up period or during first year of life according to maternal infection during the entire follow-up period and in the first year of life, compared with non-use.

	Entire follow-up period		First year of life	
	Crude incidence rate ratio (95% CI)	Adjusted* incidence rate ratio (95% CI)	Crude incidence rate ratio (95% CI)	Adjusted* incidence rate ratio (95% CI)
Antibiotic prescription				
Anytime during pregnancy	1·46 (1·27–1·66)	1·40 (1·22–1·61)	1·46 (1·17–1·83)	1·44 (1·14–1·81)
First trimester	1·67 (1·39–2·00)	1·58 (1·32–1·90)	1·47 (1·07–2·02)	1·41 (1·02–1·96)
Second trimester	1·44 (1·20–1·72)	1·37 (1·14–1·64)	1·63 (1·23–2·16)	1·57 (1·17–2·10)
Third trimester	1·48 (1·23–1·78)	1·41 (1·17–1·71)	1·45 (1·07–1·97)	1·41 (1·03–1·94)
Hospital diagnosis of infection	1·51 (1·09–2·10)	1·36 (0·97–1·90)	1·36 (0·78–2·39)	1·30 (0·74–2·29)
Number of antibiotic prescriptions during pregnancy				
1	1·38 (1·18–1·62)	1·34 (1·14–1·57)	1·40 (1·08–1·82)	1·40 (1·07–1·83)
2	1·34 (1·04–1·73)	1·31 (1·01–1·70)	1·49 (0·99–2·22)	1·50 (1·00–2·25)
3	2·11 (1·50–2·96)	1·97 (1·39–2·79)	1·56 (0·82–2·93)	1·40 (0·72–2·73)
>3	2·01 (1·32–3·08)	1·81 (1·17–2·80)	2·00 (1·03–3·90)	1·77 (0·87–3·59)
P for linear trend	0·02	0·07	0·3	0·6

*Adjusted for birth year, birth order (1, 2+), maternal age (≤20, 21–34, ≥35), smoking in pregnancy (yes/no), maternal, history of epilepsy, and maternal history of diabetes or preeclampsia/eclampsia.

In the analysis of epilepsy types, adjusted IRRs associated with prenatal exposure to maternal infection were 1.53 (95% CI: 1.18–1.98) for partial epilepsy and 1.39 (95% CI: 1.09–1.79) for generalised epilepsy. Among the 948 children diagnosed with epilepsy, 612 (65%) had a prescription for an antiepileptic drug. The IR of antiepileptic-drug-treated epilepsy was 78 per 100 000 PY among children prenatally exposed to either maternal antibiotic treatment or maternal hospitalisation with infection. The corresponding IR was 51 among the unexposed children. This yielded a crude IRR of 1.51 (95% CI: 1.28–1.78) and an adjusted IRR of 1·47 (95% CI: 1.24–1.73).

Among children whose mothers had a history of epilepsy, the IR for epilepsy was 260 per 100 000 PY and the adjusted IRR associated with prenatal exposure to maternal infection was 2.61 (95% CI: 1.15–5.92). The association between maternal infection and epilepsy did not vary according to any other perinatal characteristics examined and did not change when the analyses were restricted to children without congenital malformations (data not shown; estimates are available from the authors upon request).

## Discussion

In this population-based historical cohort study, we found a 40% increased risk of epilepsy associated with prenatal exposure to maternal infection. This association could be caused by the infection itself, its antecedents or consequences, or by antibiotic treatment. However, the similar magnitude of IR observed for different types of antibiotics argues against the role of specific types of infections or specific treatments. Our study extends the findings by Sun *et al*,. based on data from the Danish National Birth Cohort (1996–2002) [27], that maternal infections of different types, measured by self-report collected twice during pregnancy and additionally at 6 months after delivery, were associated with epilepsy in offspring [11]. While the Sun *et al.* study examined a nationwide sample of births in Denmark in which participation was based on self-selection and our study encompassed all births in the two northern regions of Denmark, we cannot rule out at least some overlap between the two study populations during 1999–2002. Given the similarity of the settings, the overall consistency of results is reassuring. Our study confirmed, using objective measures of infection, the overall association, found earlier, between maternal systemic infection and risk of epilepsy regardless of infection site. However, we found no association with antifungal treatment, while Sun *et al.*, found that self-reported maternal vaginal yeast infection was associated with more than a two-fold increased risk of childhood epilepsy. Given that most antifungals are administered locally, our finding supports the argument that systemic effects of disease or treatment underlie the association with epilepsy in offspring.

Furthermore, our finding is in line with two previous studies showing an association between intrapartum fever and increased risk of neonatal seizures [28], [29]. Another recent study based on the Danish National Birth Cohort found no association between self-reported elevated maternal body temperature (due to fever or sauna use) during pregnancy and increased risk of epilepsy in offspring [30]. However, children with prenatal exposure to more than two maternal fever episodes, maternal fever with urinary symptoms, or maternal fever of 39.0°C or did have an increased risk of epilepsy –suggesting that the underlying causes of fever rather than elevated temperature play a role [30]. Recently it has been suggested that inflammation may be involved in the mechanism linking fever and epilepsy [31]. Experimental studies in rodents have shown that inflammatory reactions in the brain can enhance neuronal excitability [32], and anti-inflammatory treatments reduce seizures in experimental models. Cytokines are key players in the modulation of neuronal excitability, as well as in leukocyte recruitment, and inflammatory central nervous system infections [31], yet little is known about their role in the pathogenesis of epilepsy. It is possible that maternal cytokine production in response to an infection during pregnancy induces foetal neurological injury [29]. Our finding of a nearly threefold increased IRR for epilepsy among children born to mothers with epilepsy suggested that infants genetically predisposed to epilepsy may be more susceptible to inflammatory reactions.

### Strengths and limitations of the study

We conducted a large study in a well-defined population and within a uniform health care system. Our use of population-based registries permitted virtually complete follow-up for both exposure and outcome [33]. A potential study weakness is our reliance on hospital diagnoses of epilepsy, of which an estimated 81% (95% CI: 75%–87%) can be confirmed by a medical-chart diagnosis according to the stringent criteria in the International League against Epilepsy classification [17]. Presence of false positive records of epilepsy in registry data may partially explain the relatively low prevalence of antiepileptic drug use among mothers and offspring with an epilepsy diagnosis (between 65% and 70% in our study vs. 85% reported in other countries [18], [34]). At the same time, children in Denmark with conditions such as rolandic epilepsy or infantile spasms are not treated universally with antiepileptic drugs [35]. However, our study yielded similar relative risk estimates for partial epilepsy and generalised epilepsy. As any diagnostic misclassification is unlikely to differ by preceding maternal infection status, the statistical expectation for the direction of a resulting bias is towards the null, unless an upward bias arises by chance [36].

We based our study on the assumption that use of antibiotics is a valid marker for infection since infections are the specific indication for antibiotics. A study in Denmark of 7606 patients who were prescribed antibiotics by one of 602 general practitioners found that only 0.7% received antibiotic prescription prophylactically, and only 0.6% of patients received such prescription for unknown or not specified infections. In the remaining 98.7% of the patients, a prescription to an antibiotic could be traced to a specific infection diagnosis [37]. A Swedish study in patients who underwent inguinal hernia repair found that the risk of antibiotic treatment during the post-surgical period was of the same order of magnitude as infection rates reported in the Swedish Hernia Register and review studies and concluded that surveillance of postoperative antibiotic use may be considered as a resource-saving surrogate marker for surgical-site infections [38].

We do not know whether the pregnant women in our study actually took the drugs prescribed to them. We speculate that compliance was likely to be high, particularly for those redeeming more than one prescription, since financial and time expenditures were involved in obtaining the medications. Any misclassification of medication use due to noncompliance would be expected to attenuate our relative estimates because it is likely unrelated to diagnosis of epilepsy in children.

In conclusion, our data showed a slightly increased risk of epilepsy associated with prenatal exposure to maternal infections. Similar risk estimates for different categories of antibiotics suggest that the increased risk stems from the consequences of infection rather than the type of infection or drug.

## Supporting Information

Appendix S1 **Hospital diagnoses (ICD-10 codes) and prescription (ATC) codes for conditions and medications measured in this study.**
(DOCX)
