# Supplementary material for: Maternal Use of Antibiotics, Hospitalisation for Infection during Pregnancy, and Risk of Childhood Epilepsy: A Population-Based Cohort Study
Source: PLoS One. 2012 Jan 25;7(1):e30850. doi: 10.1371/journal.pone.0030850 (PMC3266299; doi:10.1371/journal.pone.0030850)
Supplement: Appendix S1 — Hospital diagnoses (ICD-10 codes) and prescription (ATC) codes for conditions and medications measured in this study. (DOCX) [file pone.0030850.s001.docx]

**Appendix S1.**

| **Disease** | **ICD-10 codes** |
| --- | --- |
| Epilepsy | G40, G41 |
| Partial epilepsy | G40·0-G40·2 |
| Generalised epilepsy | G40·3-G40·4 |
| Preeclampsia or eclampsia | O14, O15 |
| Diabetes | E10-E14 |
| Infection | A00-B99, G00-G07; I00-I02; I32.0; I33, I38; I40.0; J00-J06; J10-J18; J20-J22; J36; J39.0; J85.1; J86; K35-K37; K57.0; K57.2; K57.4; K57.8; K61; K63.0; K65.0; K65.9; K67; K75.0; K75.1; K80.0; K80.3; K80.4; K81.0; K81.9; K83.0; L00-L03; L05-L08; M00; M01; M86; N10; N12; N15.1; N30; N39.0; N41; N45; N70-N77; O23 |
| Congenital malformations | Q00-Q99 (except Q90-Q99 [chromosomal abnormalities]; Q53 [undescended testis]; or Q65-Q65.6 [congenital dislocation of the hip]) |
| **Drug** | **ATC codes** |
| Systemic antibiotics | J01 |
| Penicillins excluding pivmecillinam | J01C (excluding J01CA08, J01CA11) |
| Pivmecillinam | J01CA08, J01CA11 |
| Sulfamethizole and/or trimethoprim | J01E |
| Macrolides | J01F |
| Antifungals | J02, G01A |
| Antiepileptic drugs | N03 |
| Antidiabetic drugs | A10 |
